# Positive age beliefs protect against dementia even among elders with high-risk gene

**DOI:** 10.1371/journal.pone.0191004

**Published:** 2018-02-07

**Authors:** Becca R. Levy, Martin D. Slade, Robert H. Pietrzak, Luigi Ferrucci

**Affiliations:** 1 Social and Behavioral Science Department, Yale School of Public Health, New Haven, Connecticut, United States of America; 2 Department of Internal Medicine, Yale School of Medicine, New Haven, Connecticut, United States of America; 3 Department of Psychiatry, Yale School of Medicine, New Haven, Connecticut, United States of America; 4 U.S. Department of Veterans Affairs National Center for Posttraumatic Stress Disorder, West Haven, Connecticut, United States of America; 5 Longitudinal Studies Section, National Institute on Aging, Baltimore, Maryland, United States of America; Nathan S Kline Institute, UNITED STATES

## Abstract

One of the strongest risk factors for dementia is the ε4 variant of the *APOE* gene. Yet, many who carry it never develop dementia. The current study examined for the first time whether positive age beliefs that are acquired from the culture may reduce the risk of developing dementia among older individuals, including those who are *APOE* ε4 carriers. The cohort consisted of 4,765 Health and Retirement Study participants who were aged 60 or older and dementia-free at baseline. As predicted, in the total sample those with positive age beliefs at baseline were significantly less likely to develop dementia, after adjusting for relevant covariates. Among those with *APOE ε4*, those with positive age beliefs were 49.8% less likely to develop dementia than those with negative age beliefs. The results of this study suggest that positive age beliefs, which are modifiable and have been found to reduce stress, can act as a protective factor, even for older individuals at high risk of dementia.

## Introduction

One quarter of the population carries the ε4 variant of the *APOE* gene, which is one of the strongest risk factors for dementia [1]. Yet, only 47% of *APOE* ε4 carriers develop dementia [2]. The reason the other 53% never develop dementia was unknown. The current study examined for the first time whether an environmental factor that is assimilated from the surrounding culture, positive age beliefs—or perceptions about various aspects of old age, reduces the risk of dementia for *APOE* ε4 carriers as well as older individuals in general.

Considerable research has found that positive age beliefs predict better cognitive performance; whereas, negative age beliefs predict worse cognitive performance [3, 4]. The pattern of age beliefs predicting cognition has been supported by cross-cultural [5], experimental [3, 4], and longitudinal [6–8] studies, together with three meta-analyses [3, 9, 10]. Further, a recent study found that negative age beliefs predicted the development of Alzheimer’s disease biomarkers [11].

Most prior studies examining the etiology of dementia have focused on factors that are negative (e.g., smoking) [12] or immutable (e.g., race) [13] or both (e.g., *APOE* ε4) [1, 2]. In contrast, the age beliefs on which we focused are both positive and modifiable. Short- and long-term randomized controlled interventions conducted with older participants have shown that positive age beliefs can be bolstered and negative age beliefs can be mitigated with corresponding changes in cognitive and physical performance [4, 14].

According to stereotype embodiment theory, individuals assimilate a variety of age beliefs from the culture starting at a young age and these beliefs are reinforced across the lifespan; when they become self-relevant in later life, the beliefs can become a resource for or a barrier against good health outcomes, depending on whether they are positive or negative, respectively [15]. The mechanism by which age beliefs could influence dementia likely involves stress. The evidence to support this mechanism comes from both experimental and longitudinal research. One set of studies found that negative age beliefs can exacerbate stress; in contrast, positive age beliefs can help buffer against the deleterious effects of stress [15–17]. Another set of studies suggests that stress can contribute to the development of dementia [18, 19].

The positive age beliefs of older individuals appear to provide a means of coping with exposure to ageism which is prevalent in society [9, 15]. It has been shown that older participants in a positive-age-belief intervention interpreted their environment in a more age-friendly way [14]. The reduction of stress by positive age beliefs could potentially contribute to a lower incidence of dementia among older individuals in general and specifically among those with *APOE* ε4.

Taking all of these considerations into account, we hypothesized that positive age beliefs will protect older individuals, including *APOE* ε4 carriers, from developing dementia.

## Methods

### Cohort

The Health and Retirement Study (HRS) consists of a biennial survey of a nationally representative sample of older Americans. The sample is diverse in age, ethnicity, geography, socio-economic status, education, and sex [20]. University of Michigan Institute of Social Research investigators designed and administer the survey.

Our study sample included all HRS participants for whom there were measures of age beliefs, cognition, and the covariates, and at baseline were at least 60 years old and dementia-free as assessed by their baseline Telephone Interview for Cognitive Status (TICS) [21] dementia score (see description of the TICS in Outcome: Dementia Incidence). Participants were followed for up to 4 years. As recommended by HRS investigators, we excluded participants who had *APOE* posterior probability scores < .8 [22]; these HRS-generated scores are a measure of the single nucleotide polymorphism (SNP) imputation quality used to generate *APOE* variants for each HRS participant [22]. In our sample, 99% of the participants had posterior probability scores of greater than .8. The pattern of significant results remained when we included participants with a posterior probability score of .8 or lower.

The final cohort of the current study consisted of 4,765 participants, with an average age of 72 years (*SD* = 7.19 years). Their race/ethnic composition was 91% White, 6% Black, and 3% other. Most of the sample had a high-school education or greater (86%, with 26% having completed college), was married (93%), and had a history of smoking (58%).

The *APOE* genotype of the cohort was assessed from saliva samples collected during home visits. A random half of the sample was asked for samples in 2006 and the other half in 2008. Saliva-collection rates were 83% in 2006 and 84% in 2008. Genotyping was performed by the National Institute of Health Center for Inherited Disease Research. The genetic information was archived and maintained by the National Center for Biotechnology Information. The Illumina HumanOmi1-Quad and Illumina Human Omni-2.5 Quad bead chips were used as genotyping platforms. Consistent with other studies [1], 26% of the participants were *APOE* ε4 carriers in our cohort (*n* = 1,250), and within this group variants included 85% ε4/ε3, 8% ε4/ε2, and 7% ε4/ε4.

All participants provided informed consent: verbally for telephone interviews and written for biological samples. The study procedures were approved by the University of Michigan Institutional Review Board. The secondary data analysis was approved by the Yale Human Investigation Committee.

### Predictor: Positive age beliefs

Age beliefs were assessed with the five-item Attitude toward Aging (ATA) subscale of the Philadelphia Geriatric Center Morale Scale (e.g., *The older I get*, *the more useless I feel*) [23, 24]. Potential responses range from *strongly disagree* to *strongly agree*. We reverse-scored responses, so that total scores ranged from 5 to 30, with a higher score indicating more-positive age beliefs. The scale has good internal and external validity [15, 23, 24]. The ATA was administered in 2008 to a random half of the HRS participants and to the other half in 2010 [25].

### Outcome: Dementia incidence

Dementia was measured with the TICS, which assesses a range of cognitive domains including short-term memory, delayed recall, and mathematical skills [21, 26]. It was administered every 2 years [26]. TICS is valid and reliable in assessing cognitive function in older adults of similar education and age as our current sample, shows little evidence of ceiling and/or practice effects, and has good sensitivity and specificity for identifying dementia [27–32]. Langa and colleagues have validated the TICS cut-points which classify scores of 0 to 6 as dementia, 7 to 11 as cognitive impairment no dementia, and 12 or above as normal cognition [31]. These TICS cut-points have been successfully validated with a 3-to-4-hour comprehensive neuropsychological and clinical assessment with older individuals [27, 31]. To avoid practice effects, the HRS investigators created four non-overlapping word lists that are given in each of four subsequent waves [26].

In order to identify new cases of dementia, participants were included in the study if at baseline they were classified as not having dementia (TICS score of 7 or higher), according to the established TICS cut-point score [27, 31]. Dementia incidence was then established by participants dropping to the TICS dementia cut-point score of 6, or lower, in one of the waves following baseline.

### Covariates

Covariates that research has shown relate to age beliefs and/or dementia [1, 11–15, 19] consisted of baseline age, sex, race (White, Black, and other), education, marital status, smoking history, depression as assessed by the Center for Epidemiologic Studies Depression Scale (CES-D) [33], cognitive performance as assessed by the TICS [21], *APOE* ε4 status, and whether a health-care provider had told participants they have cardiovascular disease and/ or diabetes. A higher baseline performance on TICS significantly related to positive age beliefs, r = .19, p < .001. The covariates of age and TICS [21] were included as continuous variables. Education was included as high-school education or greater, marital status was included as married or not, smoking history was included as ever smoked or not. CES-D was included as a dichotomous variable based on the frequently used cut-off score of 16 or greater to indicate individuals at risk for clinical depression [34]. *APOE* ε4 status, which was only included in the model with the total sample, but not the *APOE* ε4 carrier model, was included as *APOE* ε4-carrier or not.

### Statistical analysis

To examine the hypothesis that positive age beliefs protect older individuals in the full sample from developing dementia, we conducted a prospective logistic regression model over 4 years. The model was conducted with the earliest-administered age-belief measure as the predictor (examined as a continuous variable) and incident dementia as the outcome, among all participants who were 60 years or older and dementia-free at baseline. We conducted a backward elimination covariate strategy in this model, while forcing the inclusion of demographic variables (i.e., age, education, sex, and race) and health variables (i.e., cardiovascular disease, diabetes, and *APOE* status), following the covariate-inclusion strategy that was used in a previous study of the association of age beliefs with Alzheimer’s disease biomarkers [11]. The following covariates significantly increased the risk of incident dementia: age, education, race, cardiovascular disease, *APOE*, and baseline cognitive performance. Thus, the covariates included in the final models consisted of those that remained after backward elimination, and the demographic and health variables that were specified in the forced inclusion. These included age, education, sex, race, cardiovascular disease, diabetes, *APOE* ε4 status, and baseline cognitive performance.

**Fig 1:**
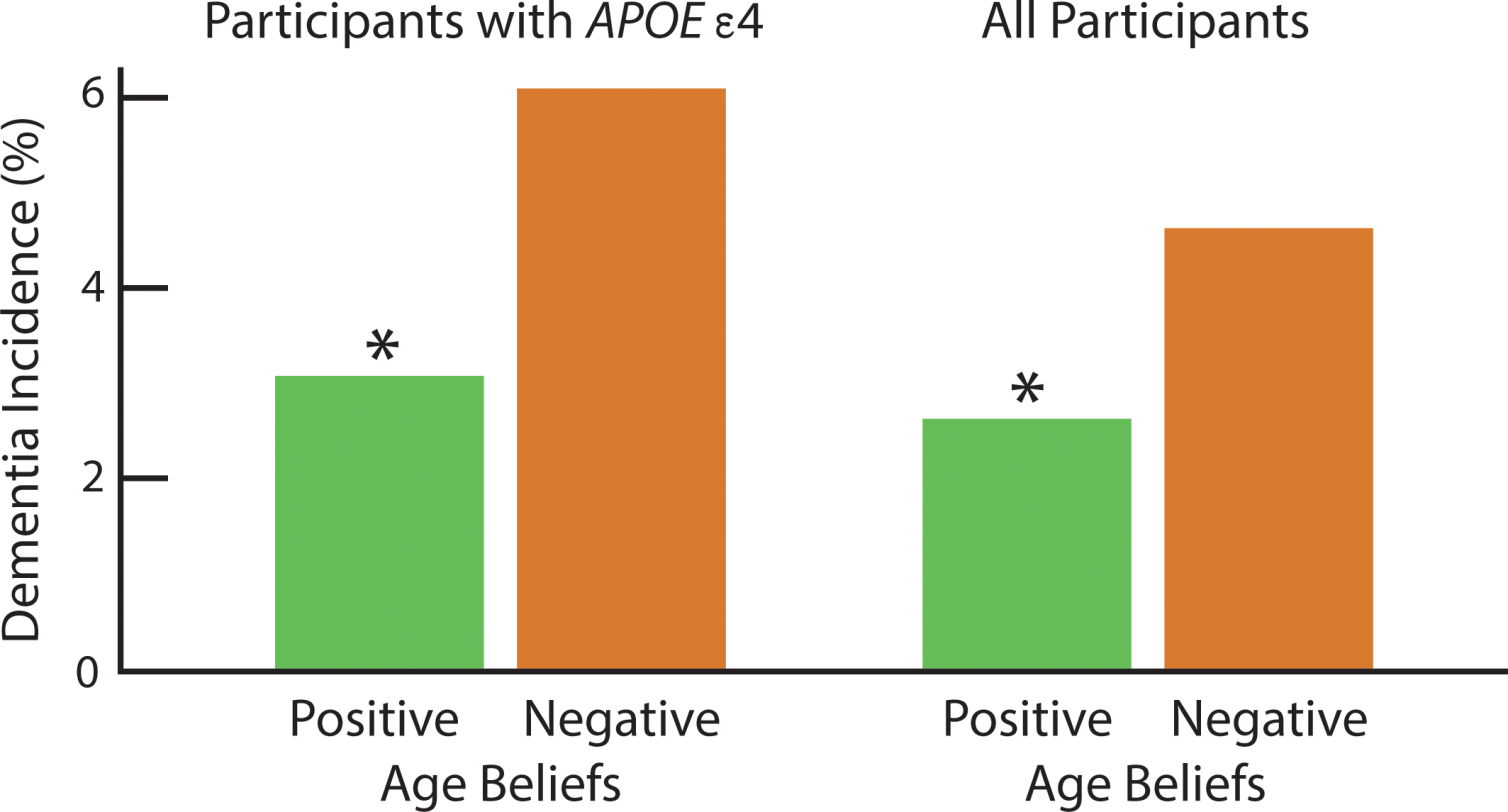
Positive age beliefs associated with resisting dementia among participants with *APOE* ε4 and all participants. Dementia incidence was assessed over 4 years and adjusted for covariates among participants who were dementia-free at baseline. The symbol * indicates significant difference at p < .05 between those with positive and negative age beliefs among participants with *APOE* ε4 and among all participants. Age-belief scores were dichotomized at the mean of 15.

To examine whether positive age beliefs also protected *APOE* ε4 carriers from developing dementia, we conducted a subset analysis. In this model, we considered whether those with more-positive age beliefs were less likely to develop dementia than those with more-negative age beliefs. Covariates were the same as in the total sample, with the exception of *APOE* status. For the graphic representation, we dichotomized age beliefs at the mean of 15 and adjusted for covariates (see Fig 1).

To examine whether positive age beliefs provide a countervailing force against the dementia-risk-factor *APOE* ε4, so that the risk of dementia among *APOE* ε4 carriers with positive age beliefs would be equal to the risk of dementia among non-carriers, we conducted a logistic regression analysis that examined whether or not the development of dementia among *APOE* ε4 carriers with positive age beliefs differed from non-*APOE* ε4 carriers with both positive and negative age beliefs.

In order to compare the contribution of variables to dementia, we standardized all variables (except those that are categorical and have more than two levels, such as race) based on our data set [35].

## Results

As predicted, positive age beliefs protected older individuals from developing dementia in the total sample, RR = .81, 95% CI = .67, .97, p = .03, adjusting for the covariates of age, education, sex, race, cardiovascular disease, diabetes, baseline cognitive performance, and *APOE* ε4 status. In the total sample, those with positive age beliefs at baseline had a 2.60% risk of developing dementia, compared to the 4.61% risk for those with negative age beliefs at baseline, during the 4-year study period (see Fig 1).

Also as predicted, positive age beliefs protected older individuals from developing dementia among *APOE* ε4 carriers, RR = .69, 95% CI = .50, .94, p = .018, adjusting for the covariates of age, education, sex, race, cardiovascular disease, diabetes, and baseline cognitive performance. Among members of the *APOE* ε4 group, those with positive age beliefs at baseline had a 2.7% risk of developing dementia, compared to the 6.14% risk for those with negative age beliefs, in the 4 years studied (see Fig 1).

There was no significant difference between dementia incidence of the *APOE* ε4 group holding positive age beliefs and individuals without *APOE* ε4, holding positive or negative age beliefs, χ 2 {\displaystyle \chi ^{2}} *χ*^2^ = .33, p = .57. In addition, the difference in dementia conversion rates for those with and without *APOE* ε4 (regardless of age beliefs) was 2.14%. That is, the dementia conversion rate of those with *APOE* ε4 was 4.50%; whereas, it was 2.36% for those without *APOE* ε4. This was similar to the difference in dementia conversion rates for those with positive and negative age beliefs (2.17%).

## Discussion

The current study provides evidence that a cultural construct, age beliefs, may contribute to the development of dementia in older individuals. In our sample, the dementia conversion rate due to negative age beliefs was within .03% of the dementia conversion rate due to *APOE* ε4, a well-established risk factor for dementia.

The impact of positive age beliefs as a protective factor against developing dementia was suggested by our finding that in the total sample participants holding these beliefs at baseline had a 43.6% lower risk of developing dementia over the course of 4 years, compared to those holding negative age beliefs at baseline. Moreover, among those with *APOE* ε4, there was a 49.8% lower risk of dementia for those holding positive age beliefs at baseline, compared to those holding negative age beliefs at baseline. These significant patterns existed after adjusting for a number of important covariates, including age and baseline cognitive performance.

The results suggest that positive age beliefs among those with *APOE* ε4 could be capable of helping to offset the influence of this genetic risk factor. For *APOE* ε4 carriers with positive age beliefs had a risk of developing dementia that is similar to the risk of their same-aged peers without *APOE* ε4, regardless of age beliefs.

Positive age beliefs may impact the same causal pathway responsible for the excess genetic risk of *APOE* ε4. If positive age beliefs lessen the influence of *APOE* ε4, it would likely occur as a later-life epigenetic process. In this process, the positive age beliefs, which can reduce stress levels [16], could alter gene expression.

Although there is the possibility that dementia influenced age beliefs in the current study, a number of factors suggest that age beliefs influenced dementia. First, the baseline measurement of these beliefs preceded the measurement of dementia by at least two years. Second, all participants were dementia-free at baseline and the models adjusted for baseline cognition. Third, experimental studies have shown that when older individuals are randomly assigned to a negative-age-stereotype intervention, corresponding to the ageism that older individuals routinely encounter in everyday life, it leads to an increase of cardiovascular reactivity to stress and reduced memory performance [4, 16]. Fourth, age beliefs tend to be internalized early in life and then remain stable over the lifespan, without interventions [36].

The age belief-gene finding has a number of possible applications. It could advance research on age beliefs, which has not previously considered how they operate in a high-risk-genetic group. Conversely, it could contribute to genetic research through the novel application of the age-belief role. Also, it could inform personalized-medicine strategies to improve cognitive health by identifying those who are at higher risk of developing dementia, as indicated by age beliefs, and then by bolstering positive age beliefs through an intervention. More generally, our finding could provide a rationale for a public-health campaign to combat the societal sources of negative age beliefs.

## References

[1] Beydoun MA, Boueiz A, Abougergi MS, Kitner-Triolo MH, Beydoun HA, Resnick SM, et al Sex differences in the association of the apolipoprotein E epsilon 4 allele with incidence of dementia, cognitive impairment, and decline. Neurobiol Aging. 2012;33: 720–731. doi: 10.1016/j.neurobiolaging.2010.05.017 20619505 10.1016/j.neurobiolaging.2010.05.017 PMC2974952

[2] Liu CC, Kanekiyo T, Xu H, Bu G. Apolipoprotein E and Alzheimer disease: Risk, mechanisms and therapy. Nat Rev Neurol. 2013;9: 106–118. doi: 10.1038/nrneurol.2012.263 23296339 10.1038/nrneurol.2012.263 PMC3726719

[3] Lamont RA, Swift HJ, Abrams D. A review and meta-analysis of age-based stereotype threat: Negative stereotypes, not facts, do the damage. Psychol Aging. 2015;30: 180–193. doi: 10.1037/a0038586 25621742 10.1037/a0038586 PMC4360754

[4] Levy B. Improving memory in old age through implicit self-stereotyping. J Pers Soc Psychol. 1996;71: 1092–1107. 8979380 10.1037//0022-3514.71.6.1092

[5] Levy B, Langer E. Aging free from negative stereotypes: Successful memory in China and among the American Deaf. J Pers Soc Psychol. 1994;66: 989–97. 8046582 10.1037//0022-3514.66.6.989

[6] Levy BR, Zonderman AB, Slade MD, Ferrucci L. Memory shaped by age stereotypes over time. J Gerontol B Psychol Sci Soc Sci. 2012;67: 432–436. doi: 10.1093/geronb/gbr120 22056832 10.1093/geronb/gbr120 PMC3391075

[7] Robertson DA, King-Kallimanis BL, Kenny RA. Negative perceptions of aging predict longitudinal decline in cognitive function. Psychol Aging. 2016;31: 71–81. doi: 10.1037/pag0000061 26691302 10.1037/pag0000061

[8] Seidler AL, Wolff JK. Bidirectional associations between self-perceptions of aging and processing speed across 3 years. GeroPsych. 2017;30: 49–59.

[9] Meisner BA. A meta-analysis of positive and negative age stereotype priming effects on behavior among older adults. J Gerontol B Psychol Sci Soc Sci. 2012;67: 13–17. doi: 10.1093/geronb/gbr062 21746872 10.1093/geronb/gbr062

[10] Horton S, Baker J, Pearce GW, Deakin JM. On the malleability of performance: Implications for seniors. J of Applied Gerontol. 2008;27: 446–465.

[11] Levy BR, Ferrucci L, Zonderman AB, Slade MD, Troncoso J, Resnick SM. A culture-brain link: Negative age stereotypes predict Alzheimer's disease biomarkers. Psychol Aging. 2016;31: 82–88. doi: 10.1037/pag0000062 26641877 10.1037/pag0000062 PMC4853823

[12] Anstey KJ, von Sanden, Salim A, O'Kearney R. Smoking as a risk factor for dementia and cognitive decline: A meta-analysis of prospective studies. Am J Epidemiol. 2007;166: 367–378. doi: 10.1093/aje/kwm116 17573335 10.1093/aje/kwm116

[13] Gilsanz P, Mayeda ER, Glymour MM, Quesenberry CP, Whitmer RA. Association between birth in a high stroke mortality state, race, and risk of dementia. JAMA Neurol. 2017;74: 1056–1062. doi: 10.1001/jamaneurol.2017.1553 28759663 10.1001/jamaneurol.2017.1553 PMC5691590

[14] Levy BR, Pilver C, Chung PH, Slade MD. Subliminal strengthening: Improving older individuals' physical function over time with an implicit-age-stereotype intervention. Psychol Sci. 2014;25: 2127–2135. doi: 10.1177/0956797614551970 25326508 10.1177/0956797614551970 PMC4372155

[15] Levy B. Stereotype embodiment: A psychosocial approach to aging. Curr Dir Psychol Sci. 2009;18: 332–336. 20802838 10.1111/j.1467-8721.2009.01662.x PMC2927354

[16] Levy BR, Hausdorff JM, Hencke R, Wei JY. Reducing cardiovascular stress with positive self-stereotypes of aging. J Gerontol B Psychol Sci Soc Sci. 2000; 55: 205–213. 10.1093/geronb/55.4.p205 11584877

[17] Levy BR, Moffat S, Resnick SM, Slade MD, Ferrucci L. Buffer against cumulative stress: Positive age stereotypes predict lower cortisol across 30 years. Geropsych. 2016; 29: 141–146.

[18] Yaffe K, Vittinghoff E, Lindquist K, Barnes D, Covinsky KE, Neylan T, et al Post-traumatic stress disorder and risk of dementia among US veterans. Arch Gen Psychiatry. 2010;67: 608–613. doi: 10.1001/archgenpsychiatry.2010.61 20530010 10.1001/archgenpsychiatry.2010.61 PMC2933793

[19] McEwen BS. Neurobiological and systemic effects of chronic stress. Chronic Stress. 2017;1, doi: 10.1177/2470547017692328 28856337 10.1177/2470547017692328 PMC5573220

[20] National Institute on Aging, *Growing Older in America*: *The Health and Retirement Study*. 2007, National Institutes of Health: Washington, DC.

[21] Brandt J, Spencer M, Folstein M. The telephone interview for cognitive status. Neuropsychol Behav Neurol. 1988;1: 111–117.

[22] Faul J, Smith J, Zhao W. *Health and Retirement Study*: *Candidate genes for cognition/ behavior* 2014, University of Michigan: Ann Arbor, MI.

[23] Liang J, Bollen KA The structure of the Philadelphia Geriatric Center Morale scale: A reinterpretation. J Gerontol. 1983;38: 181–189. 6827034 10.1093/geronj/38.2.181

[24] Lawton MP. The Philadelphia Geriatric Center Morale Scale: A revision. J Gerontol. 1975;30: 85–89. 1109399 10.1093/geronj/30.1.85

[25] Smith J, Fisher GG, Ryan LH, Clarke PJ, House J, Weir D. *Psychosocial and Lifestyle Questionnaire 2006–2010 Documentation Report*. 2013 Institute for Social Research: University of Michigan: Ann Arbor, Michigan.

[26] Fisher GG, Hassan J, Faul JD Rogers W, Weir DR. *Health and Retirement Study*. Imputation of cognitive measures: 1992–2014. 2017 Institute for Social Research: University of Michigan: Ann Arbor, Michigan.

[27] Crimmins EM, Kim JK, Langa KM, Weir DR. Assessment of cognition using surveys and neuropsychological assessment: The Health and Retirement Study and the Aging, Demographics, and Memory Study. J Gerontol B Psychol Sci Soc Sci. 2011;66 Suppl 1: 162–171. 10.1093/geronb/gbr048 PMC3165454 21743047

[28] Seo EH, Lee DY, Kim SG, Kim KW, Kim DH, Kim BJ et al Validity of the telephone interview for cognitive status (TICS) and modified TICS (TICSm) for mild cognitive imparment (MCI) and dementia screening. Arch Gerontol Geriatr. 2011;52: e26–e30. doi: 10.1016/j.archger.2010.04.008 20471701 10.1016/j.archger.2010.04.008

[29] Fong TG, Fearing MA, Jones RN, Shi P, Marcantonio ER, Rudolph JL, et al Telephone interview for cognitive status: Creating a crosswalk with the Mini-Mental State Examination. Alzheimers Dement. 2009;5: 492–497. doi: 10.1016/j.jalz.2009.02.007 19647495 10.1016/j.jalz.2009.02.007 PMC2783323

[30] Castanho TC, Amorim L, Zihl J, Palha JA, Sousa N, Santos NC. Telephone-based screening tools for mild cognitive impairment and dementia in aging studies: A review of validated instruments. Front Aging Neurosci. 2014;6:16 doi: 10.3389/fnagi.2014.00016 24611046 10.3389/fnagi.2014.00016 PMC3933813

[31] Langa KM, Larson EB, Karlawish JH, Cutler DM, Kabeto MU, Kim SY, Rosen AB. Trends in the prevalence and mortality of cognitive impairment in the United States: Is there evidence of a compression of cognitive morbidity? Alzheimers Dement. 2008;4: 134–144. doi: 10.1016/j.jalz.2008.01.001 18631957 10.1016/j.jalz.2008.01.001 PMC2390845

[32] Ferrucci L, Del Lungo I, Guralnik JM, Bandinelli S, Benvenuti E, Salani B, et al Is the telephone interview for cognitive status a valid alternative in persons who cannot be evaluated by the Mini Mental State Examination? Aging (Milano). 1998;10: 332–338. 9825025 10.1007/BF03339796

[33] Radloff LS. The CES-D Scale: A self-report depression scale for research in the general population. Applied Psychol Measures. 1977;1: 385–401.

[34] Lewinsohn PM, Seeley JR, Roberts RE, Allen NB. Center for Epidemiological Studies-Depression Scale (CES-D) as a screening instrument for depression among community-residing older adults. Psychol Aging. 1997;12: 277–287. 9189988 10.1037//0882-7974.12.2.277

[35] Hayes AF. Introduction to mediation, moderation, and conditional process analysis: A regression-based approach. 2013. Guilford. New York.

[36] Levy BR, Slade MD, Chung P, Gill TM. Resiliency over time of elders’ age stereotypes after encountering stressful events. J Gerontol B Psychol Sci Soc Sci. 2014;79: 886–890. 10.1093/geronb/gbu082 PMC4612340 24997287

